# Hospital-based case control study and animal study on the relationship between nonylphenol exposure and depression

**DOI:** 10.7717/peerj.11384

**Published:** 2021-05-18

**Authors:** Ya Luo, Weihong Xu, Wei Ou, Ting Wang, Changwei Yang, Songying Xie, Jie Yu, Jie Xu

**Affiliations:** 1 School of Public Health, Zunyi Medical University, Zunyi, Guizhou, PR China; 2 Department of Medicopsychology, School of Management, Zunyi Medical University, Zunyi, Guizhou, PR China; 3 Department of Nuclear Medicine, Affiliated Hospital of Zunyi Medical University, Zunyi, Guizhou, PR China

**Keywords:** Nonyphenol, Depression, Monoamine, Neurotrnsmitters, Brain-derived neuro-trophic fator

## Abstract

**Objectives:**

The aim of this work is to explore the association between chronic exposure to nonylphenol (NP), a representative environmental endocrine disruptor (EED), and the risk of depression and its potential mechanism.

**Methods:**

A hospital-based case control study was conducted from August to December 2018. Forty-one patients with confirmed depression and 47 healthy volunteers were recruited. In vitro, 20 rats were randomly divided into the control group (corn oil) and NP exposure group (*n*=10 per group), which were gavaged at a dose of 4 mg/kg w/day for 180 days.

**Results:**

The depressed patient group had higher Zung Self-Rating Depression Scale (SDS) (*P*<0.001) and Self-Rating Anxiety Scale (SAS) (*P*<0.001) scores than the healthy group. The serum NP level (*P*=0.009) in the depressed group was higher than that in the healthy group, and the BDNF level (*P*=0.001) was lower. The serum levels of monoamine neurotransmitters dopamine (DA) (*P*=0.070), epinephrine (E) (*P*=0.001), and noradrenaline (NE) (*P*=0.000) were lower than those in the healthy group. In the sucrose preference test, the sucrose preference time for the exposure group of rats was lower than that of the control group (*P*<0.001). In the forced swim test, a longer resting time was measured for the exposure group of rats as compared to the control group (*P*<0.05). The level of NP (*P*<0.001) in the brain tissue of the NP exposure group was higher than that in the control group, and the serum level of brain-derived neurotrophic factor (BDNF) (*P*=0.004) was lower. Histopathological examination of the brain biopsies illustrated that the neuronal cells and nuclei in the hippocampus of the exposed group exhibited slight shrinkage.

**Conclusion:**

Chronic exposure to NP at environmental doses will result in the accumulation of NP in the brain and blood, and induction of depression, which might be associated with the alterations in the expression levels of BDNF and monoamine neurotransmitters.

## Introduction

Depression has become a common psychiatric disorder that seriously threatens peoples physical and mental health, and it is a serious global public health problem. Depression has a high prevalence and recurrence rate, and is associated with suicide, which is the main cause of death of people aged 15-44years (Zhang, Yang & Wu, 2017). The worldwide incidence of depression has been reported to be approximately 11.0% (Liu et al., 2019) and the prevalence of depression has increased from 3.2% in 2005 to 9.61/100,000 in 2015 in China (Ren, Yu & Dong, 2020; Johnston, Powell & Anderson, 2019). There has been increasing evidence indicating that increased exposure to environmental endocrine disruptors (EEDs) is associated with increasing risk of depression (Braun et al., 2014; Harley et al., 2013; Kumar & Thakur, 2017; Quinnies et al., 2017).

Studies have shown that EEDs, such as bisphenol A (BPA) and dimethyl phthalate, can pass through the bloodbrain barrier and produce toxic effects on nerves. In animal experiments, exposure to BPA during pregnancy and lactation in female rats leads to prolonged forced swim time and depression-like behavioral changes in F1 male offspring (Borrow & Cameron, 2014). A population-based study reported that the higher level of phthalate (an EED), the higher the score of autistic features in Canadian womens offspring during pregnancy. Prenatal exposure to BPA is associated with anxiety and depression in boys aged 10-12years, and childhood exposure to BPA also impacts behavior. Exposure to EEDs during adolescence was associated with the emotional and social behavior of rats in adulthood (Jennifer etal., 2013; Frederica et al., 2016; Ying et al., 2018).

Nonylphenol (NP) is a representative EED with high bioaccumulation capacity. Currently, there is significant NP pollution in the environment. The total production is approximately 50,000t per year in China, and it is mainly used as a synthetic detergent. Our prior studies indicated that exposure to NP negatively affected learning and memory ability in F_1_ rats (Jie et al., 2010), possibly due to the alterations in the expression of c-Jun and c-Fos, and ChAT and AchE activities in hippocampus of pups (Perez-Lobato etal., 2016). However, whether exposure to NP would increase the risk of depression has not been elucidated.

Although there have been many hypotheses, the pathogenesis of depression still remains unclear. Monoamine neurotransmitters, such as dopamine, serotonin, norepinephrine, and epinephrine, have a wide range of biological activities and participate in the regulation of mood. The monoamine hypothesis, which states that decreased concentration or function of monoamine neurotransmitters is the biological basis of depression, has been relatively accepted. EEDs affect neurotransmitters and the secretion of their receptors via calcium dyshomeostasis and the interference of the kisspeptin/leptin signal pathway. Currently, there is extensive research being conducted on the environmental factors that contribute to depression (Shaina et al., 2017; Grohs et al., 2019; Kahn et al., 2020). Herein, for the first time, we analyzed the levels of NP, neurotransmitters, and brain-derived neurotrophic factor (BDNF) in patients with depression through a hospital-based case control study. Through an animal model of long-term exposure to NP, the relationship between NP and depression was analyzed, providing data regarding the pathogenesis of depression via the perspective of neurotoxicology.

## Materials and Methods

### Ethics statement

The Ethics Committee of the Zunyi Medical University approved the human study (2017-1-002) and animal study (2017-2-008). All methods were performed in accordance with guidelines and regulations of the Zunyi Medical University.

### Hospital-based case control study

#### Study design

A hospital-based case control study was conducted from August to December 2018. A total of 54 patients were diagnosed with confirmed depression for the first time in the Psychological Clinic of the Affiliated Hospital of Zunyi Medical University. Forty-one patients were recruited into the study, and 13 patients (24%) were excluded from this study due to inadequate information. Forty-seven healthy people volunteered for the survey in the Psychological Clinic of the Affiliated Hospital and were recruited to the control group. All participants received the Zung Self-Rating Depression Scale (SDS) and the Self-Rating Anxiety Scale (SAS) and provided their individual scores. The depressed patients scored above 50 for both scales, but the healthy volunteers scored below 50. An investigator blinded to the group allocation evaluated the SDS and SAS scores. All participants were required to sign an informed consent form.

#### Sample collection

Blood (8-10 ml) was collected from each participant, and subpackaged in two negative pressure tubes. One tube loaded with 45 mL of blood was centrifuged at 2,000rpm, and then stored at 80C for detection of serum EEDs, and the other one was sent to the Department of Nuclear Medicine, Affiliated Hospital of Zunyi Medical University, for detection of serum monoamine neurotransmitters and serotonin.

#### Extraction and measurement of serum NP

Approximately 0.5 mL of serum was vortexed with 4 ml of n-hexane-ether extractant (volume ratio of 7:3) for 30 s, and was then maintained at room temperature for 15 min. The supernatant was dried with nitrogen gas at 30C, further dissolved with 0.5 mL of acetonitrile, vortexed for 10 s, and transferred to high-performance liquid chromatography (HPLC) vials. HPLC conditions: excitation wavelength: 275 nm, emission wavelength: 312 nm, mobile phase: 85:15 volume ratio of acetonitrile and 0.1% glacial acetic acid, injection: 10 L, and flow rate: 1 ml/min, using a fluorescence detector.

#### Detection of serum monoamine neurotransmitters (epinephrine (E), noradrenalin (NE), dopamine (DA)) and 5-hydroxytryptophane (HTP)

Sample preparation, extraction, and acylation were performed with a **-radioimmunocounter for 1 min in accordance with the kit instructions.

#### Detection of serum brain-derived neurotrophic factor (BDNF)

Enzyme-linked immunosorbent assay (ELISA) was used to detect BDNF in serum. Serum was thawed to room temperature, and diluted 20 times. Sampling, incubation, washing, and reading optical density (OD) values were performed according to the kit instructions. A standard curve was drawn with the concentration of the standard solution as the *x*-axis, and OD value as the *y*-axis. Linear regression analysis was carried out to obtain the equation of *y*=0.0116*x*+0.074. The concentration was calculated using the OD value of the sample.

### Animal experiments

#### Experimental animals and grouping

Four-week-old Sprague-Dawley (SD) male rats were purchased from Changsha Tianqin Biotechnology Co., Ltd. and raised in a clean-grade animal room. Food and water were provided ad libitum at temperature of 242C and humidity of 50%10%. After one week of acclimation, 20 rats were randomly divided into the control group and exposure group (*n*=10 per group) according to body weight, while the simple random sampling method was used to select and group the research rats for each treatment. With the body weight of the rats ordered from the largest to the smallest, the odd number was the control group and the even number was the treatment group. After randomization, an experimenter blinded to the group allocation evaluated the rats for praxiological and morphological examination.

The exposure group was administered an NP dose of 4 mg/kgw/day by gavage, and the control group was gavaged with the same volume of corn oil. After 180 days of exposure, the rats were anesthetized with urethane, and the brains were harvested from the abdominal aorta.

### Praxiology

#### Sugar preference test

Two bottles containing 500 mL pure water and 1% (w/v) sucrose water were hung in each rat cage at the same time. The positions of the bottles were exchanged at 6 h, and the sucrose bottle was removed after 12 h to calculate the sucrose preference time.

#### Forced swim test

A forced swim test was performed at 183 days after exposure. The rats were placed in a transparent plexiglass cylinder filled with water, and the immobility time in water in 5 min was recorded.

#### Brain morphology examination

The hippocampal rat brain tissues were fixed in 10% paraformaldehyde, embedded in paraffin, sectioned, stained with hematoxylin-eosin (HE), and observed using a light microscope.

#### Measurement of levels of NP, neurotransmitters, and BDNF

The methods used for measurement were the same as those used for humans.

#### Statistical analysis

SPSS 18.0 software was used to analyze the data. The quantitative data are expressed as the meanstandard deviation or median and interquartile range (IQR). When analyzing the difference between the groups at the same exposure time, if the data were in accordance with the normal distribution, independent-samples *t*-test was used. If the data did not comply with the normal distribution, a non-parametric test (MannWhitney *U*-test) was used for comparison. The data with normal distribution were analyzed by Spearman correlation analysis. Statistical graphs were produced by GraphPad Prism 6. The test level was set at 0.05, *P*<0.05 was considered statistically significant, and all *P* values were two-sided.

## Results

### Human experiments

#### Basic conditions

A total of 41 patients with depression was enrolled in this study, with an average age of 18.413.9years. There were 47 healthy subjects in the control group, with an average age of 20.101.9years. Approximately 52% (*n*=46) of the participants (*n*=88) were female. No difference in age or sex between the two groups was found.

#### Comparison of SDS and SAS scores between the depressed and healthy groups

The depressed group had higher SDS (*P*<0.001) and SAS (*P*<0.001) scores than the healthy group (Table 1).

**Table 1:** Comparison in SDS and SAS scores between the depressed and healthy two groups ($\overline{x}\pm s$).^*^

Variables	Healthy group (*n*=41)	Depressed group (*n*=47)	*P* value
Age (year)	19.971.67	18.903.94	0.092^#^
SDS	43.3311.91	71.189.81	0.000^#^
SAS	42.588.34	61.909.82	0.000^#^

**Notes.**

* $\overline{x}$ denotes the mean, *s* denotes standard deviation, and # vs the control group.

#### Comparison of serum NP and BDNF between the depressed and healthy groups

**Figure 1:**
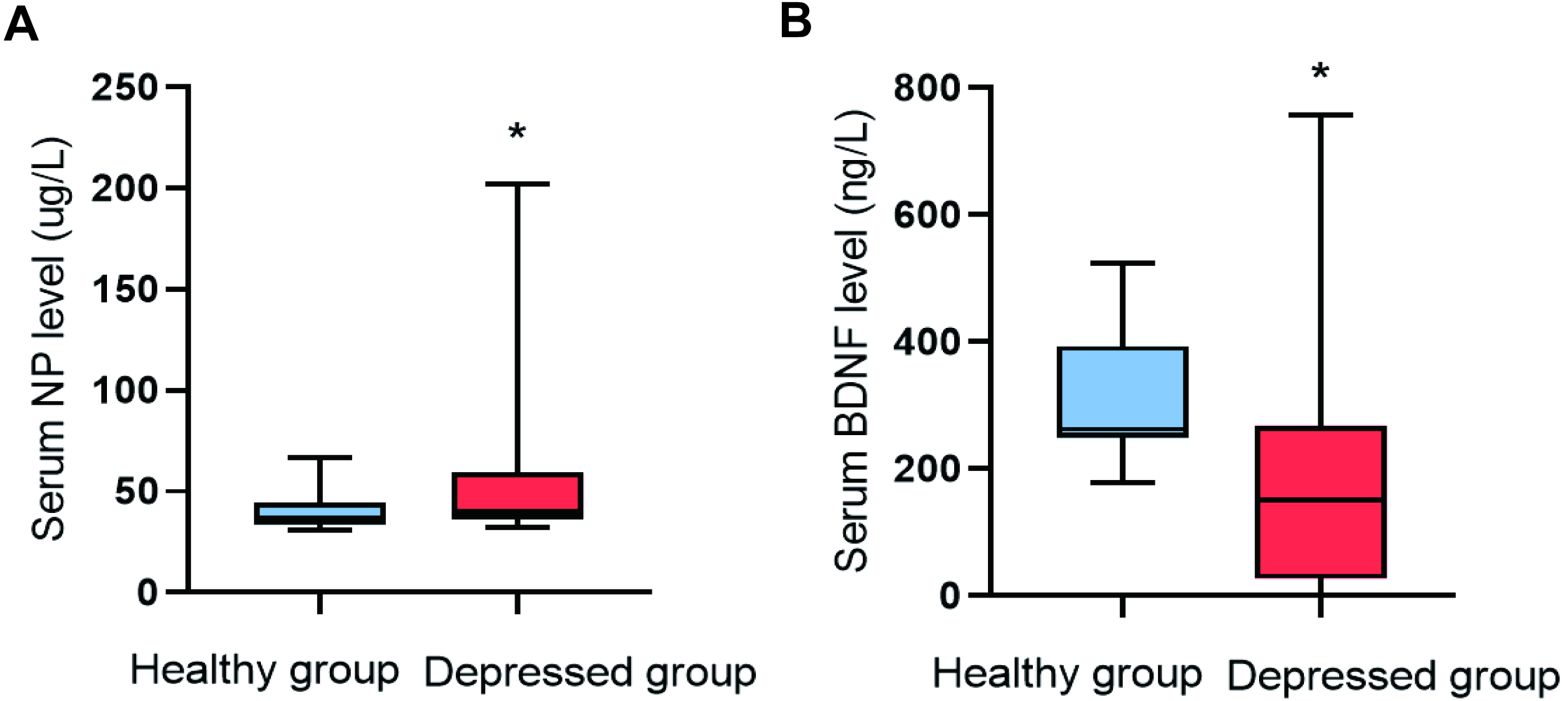
Comparison of NP and BDNF between the two groups (*n*=88). (A) Serum NP level. (B) Serum BDNF level.

The serum NP level (*P*=0.009) in the depressed group was higher than that of the healthy group, and the serum BDNF level (*P*=0.001) was lower (Fig. 1).

#### Comparison of serum monoamine neurotransmitters

**Figure 2:**
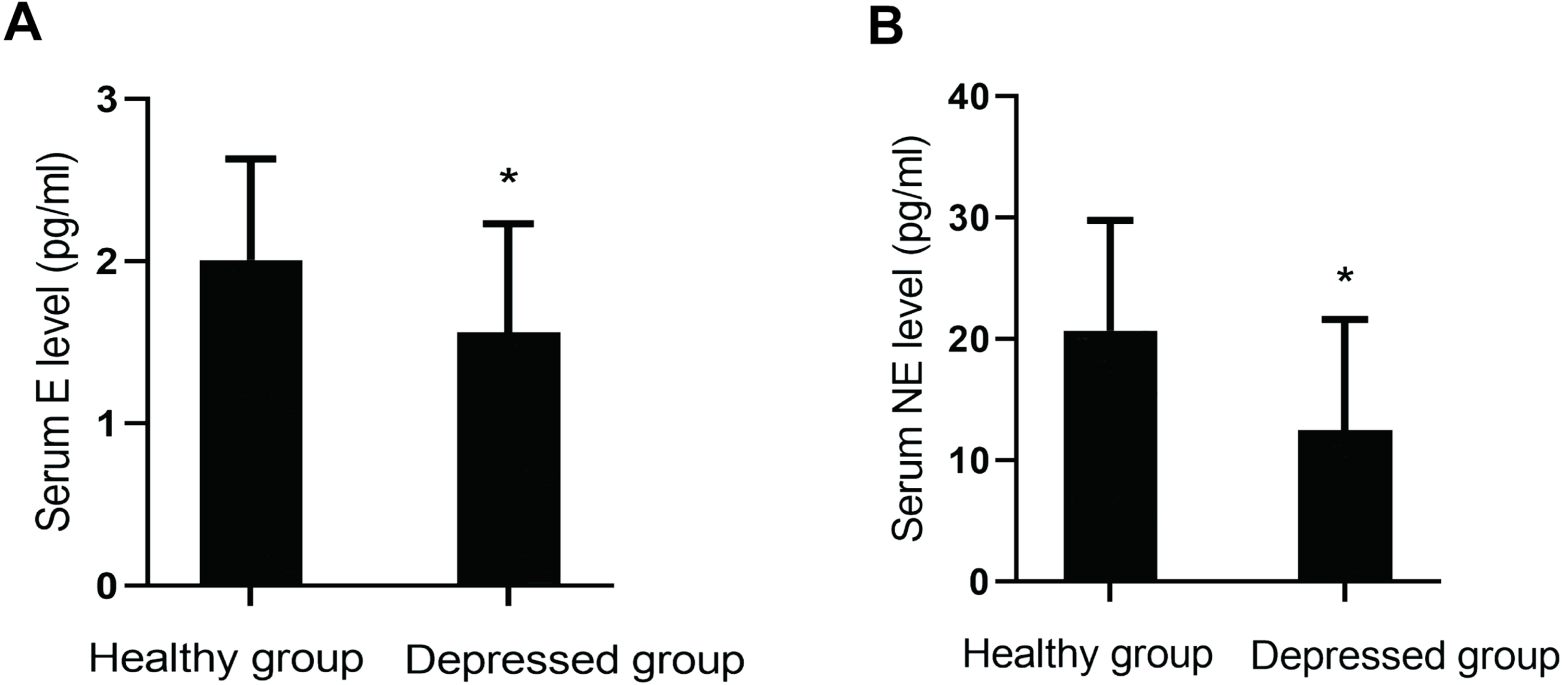
Comparison of serum monoamine neurotransmitter levels between the two groups. (A) Serum E level between the two groups. (B) Serum NE level between the two groups.

Serum levels of monoamine neurotransmitters (DA, E, NE and 5-HT) were compared between the two groups. Levels of E (*P*=0.001) and NE (*P*=0.000) in the depressed group were lower than those in the healthy group. However, there were no significant differences in the levels of 5-HT (*P*=0.762) and DA (*P*=0.070) between them (Fig. 2).

#### Spearman correlation analysis of depression score with levels of serum NP and monoamine neurotransmitters

**Figure 3:**
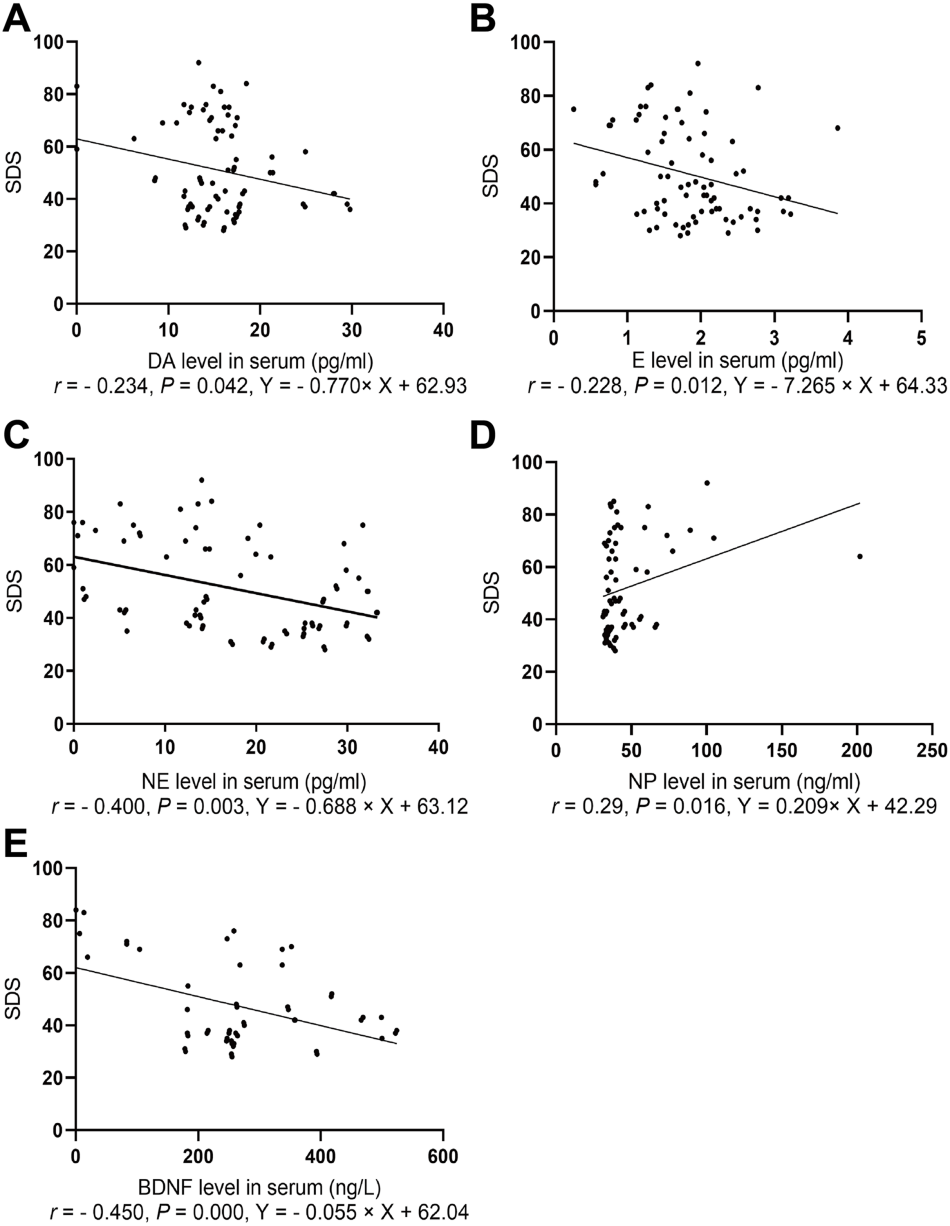
Spearman correlation analysis of SDS with NP, BDNF, and neurotransmitters. (A) The association between SDS and serum DA level. (B) The association between SDS and serum E level. (C) The association between SDS and serum NE level. (D) The association between SDS and serum BDNF level. (E) The association between SDS and serum NP level.

The depression scores in both groups were positively correlated with NP level (*r* = 0.29, *P*=0.016) and negatively correlated with levels of E (*r*=0.228, *P*=0.012), NE (*r*=0.400, *P*=0.003), and BDNF (*r*=0.450, *P*=0.000, Fig. 3).

#### Spearman correlation analysis of anxiety score with levels of serum NP, monoamine neurotransmitters, and BDNF

**Figure 4:**
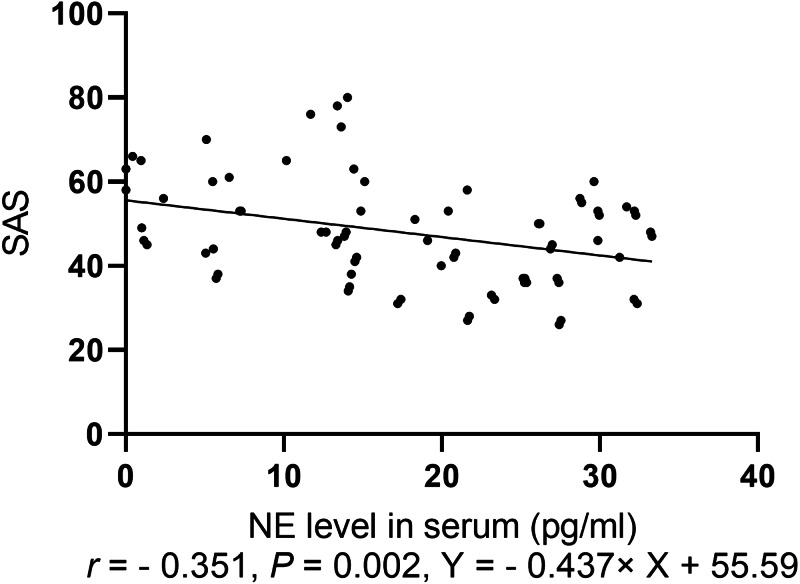
Spearman correlation analysis of SAS with levels of neurotransmitters.

Spearman correlation analysis of the anxiety score was conducted with levels of NP, monoamine neurotransmitters, and BDNF. The anxiety scores for the two groups were negatively correlated with NE level (*r*=0.351, *P*=0.002, Fig. 4).

### Animal experiments

#### Sucrose preference test

**Figure 5:**
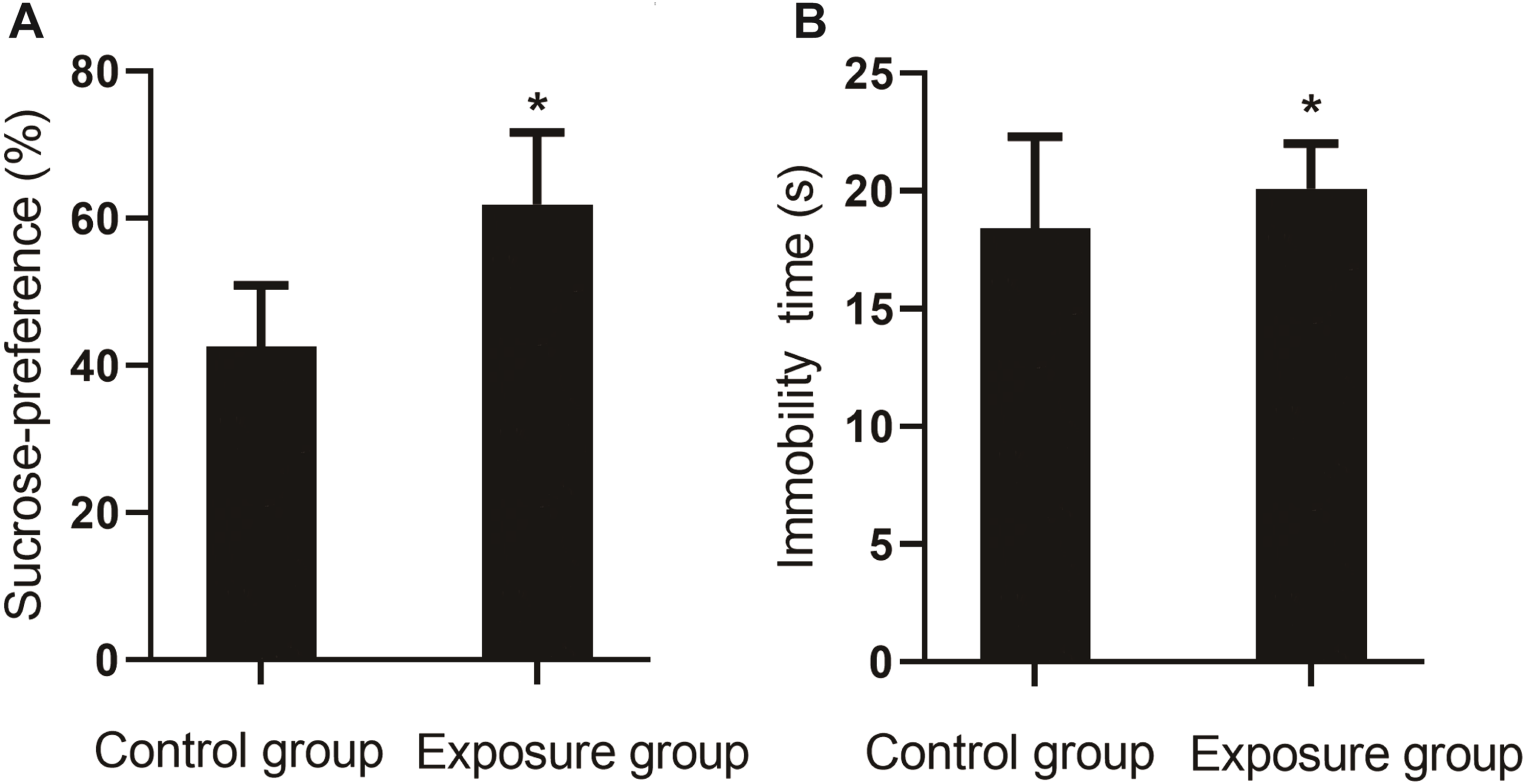
Comparison of the sucrose preference between NP-exposed and control groups in the sucrose preference test (*n*=10, $\bar {x}\pm s$). (A) The percentage of sucrose preference. (B) Immobility time. Sucrose preference of rats as determined by the sucrose preference test (*n*=10).

In the sucrose preference test, the percentage of sucrose preference for the NP-exposed group was lower than that of the control group (*P*<0.001). In the forced swim test, a longer resting time was measured for the NP-exposed group as compared to the control group (*P*<0.05, Fig. 5).

##### Measurement of neurotransmitter levels

**Figure 6:**
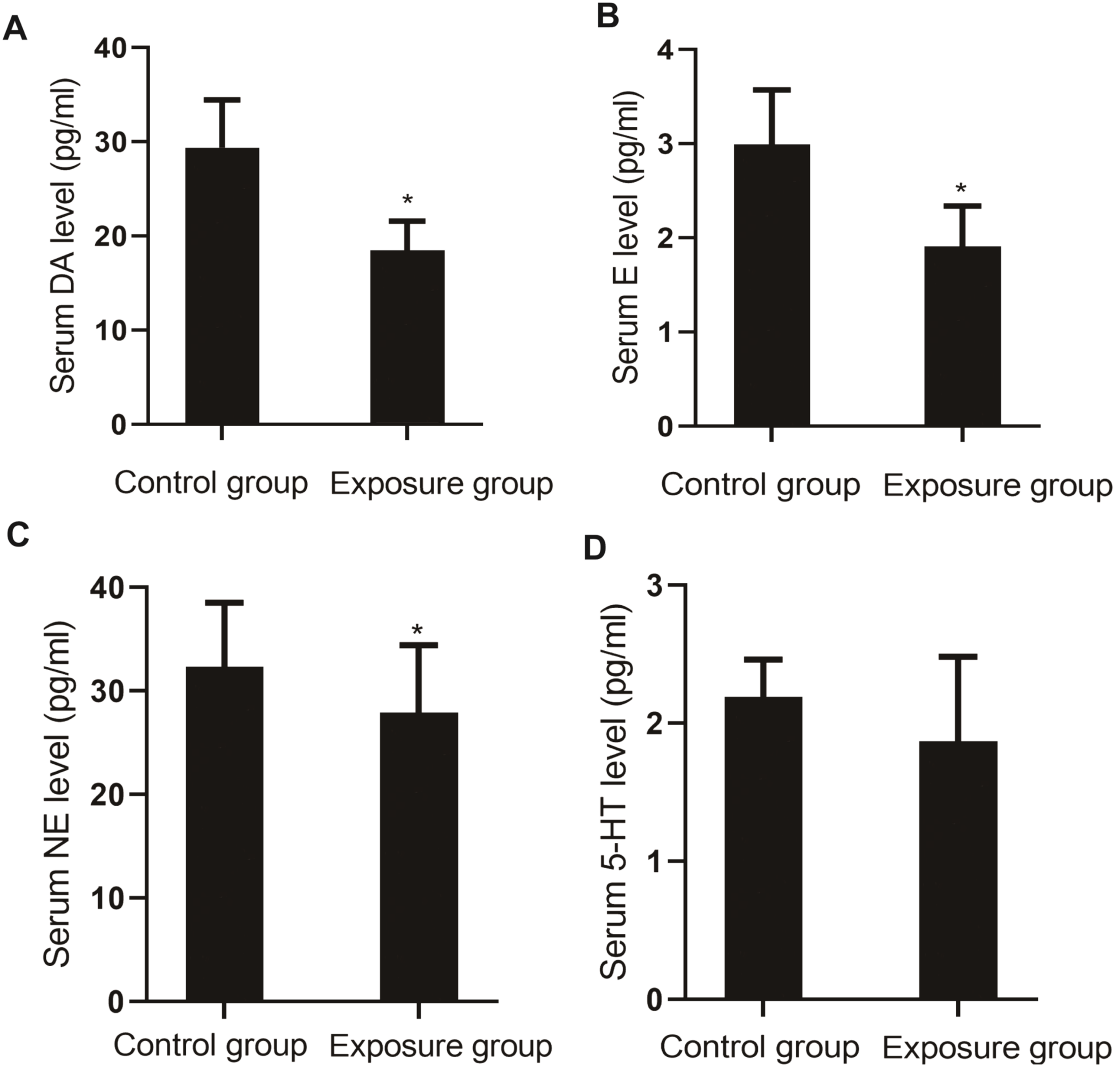
Comparison of the serum levels of monoamine neurotransmitters between the NP- exposed and control groups (*n*=10, $\bar {x}\pm s$). (A) Serum DA level between NP-exposed and control groups. (B) Serum E level between NP-exposed and control groups. (C) Serum NE level between NP-exposed and control groups. (D) Serum 5-HT level between NP-exposed and control groups.

The serum levels of monoamine neurotransmitters DA (*P*=0.004), E (*P*=0.003), NE (*P*=0.020), and 5-HT (*P*=0.036) in the NP-exposed group were lower than those in the control group (Fig. 6).

##### Comparison of NP in brain tissue and serum BDNF

**Figure 7:**
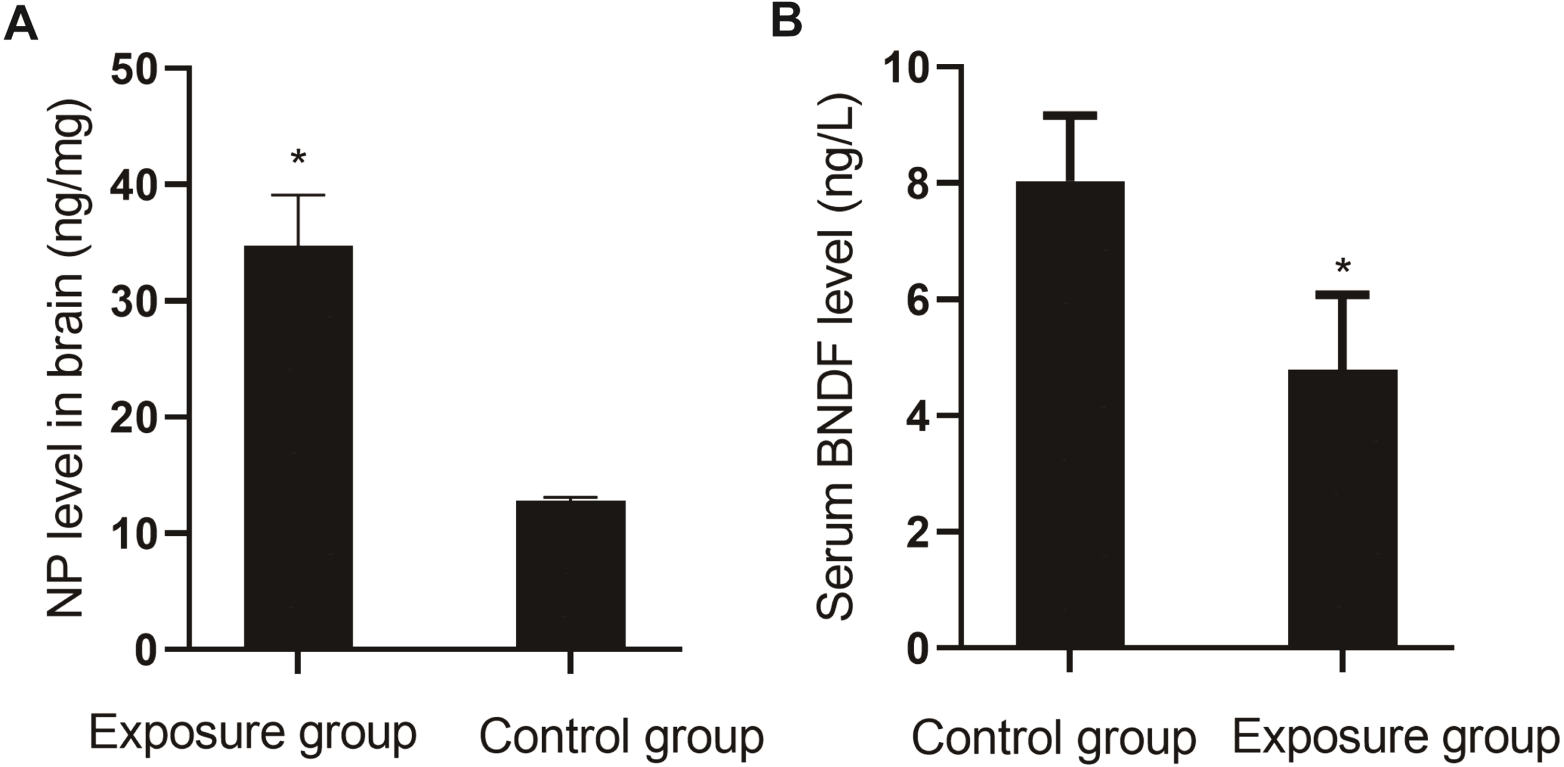
Comparison of NP levels in the brain and serum BDNF levels between the NP-exposed and control groups (*n*=10, $\bar {x}\pm s$). (A) NP level in brain between the NP-exposed and control groups. B. Serum BDNF level between the NP-exposed and control groups.

The levels of NP in the brain tissue and serum BDNF were compared between the NP-exposed and control groups. The level of NP (*P*<0.001) in the brain tissue of the exposure group was higher than that in the control group, and the level of BDNF (*P*=0.004) was lower (Fig. 7).

##### Pathological changes in the hippocampus of the rat brain

**Figure 8:**
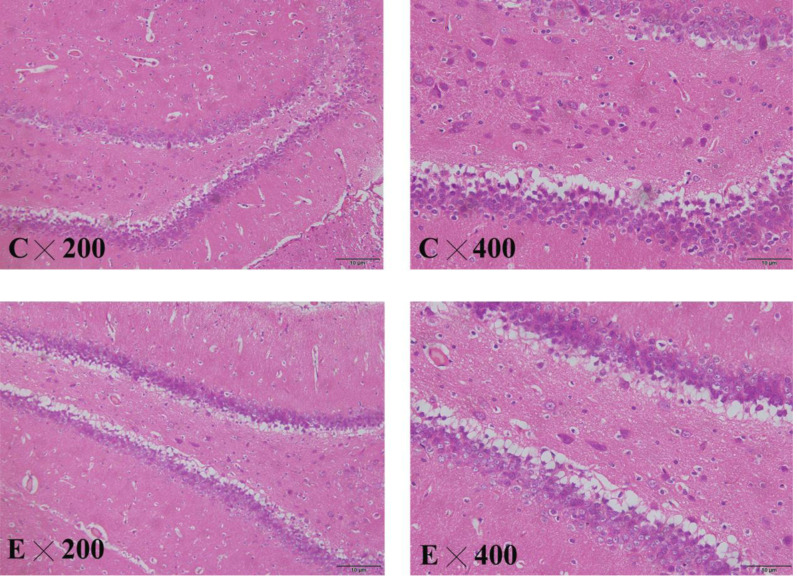
HE staining in the hippocampus of control and exposed groups. (C) Control group. (E) Exposure group.

The brain slices showed that the cytoplasm and nuclear membrane of neuronal cells in the hippocampus of the control group were clearly demarcated, with normal morphology of the nucleus. The neuronal cells and nuclei in the hippocampus of the exposed group exhibited slight shrinkage (Fig. 8).

## Discussion

Through this hospital-based case control study and animal experiments, we found that the serum NP levels in the depressed group were higher than those of the healthy group, and the levels of monoamine neurotransmitters and BDNF were lower, which was consistent with the monoamine neurotransmitter hypothesis and Hayley and Kuhlmanns study (Lee et al., 2017). After the rats were exposed to NP for 180 days, depression-like behavioral changes were determined by the sucrose preference test and forced swim test. The levels of NP in the brain tissue, serum monoamine neurotransmitters, and BDNF were measured, and the pathology of the hippocampus was observed. The NP levels in the brain tissue of rats in the exposure group were higher than that in the control group, and the serum monoamine neurotransmitters and BDNF levels were lower. The results of animal experiments were consistent with those of human surveys (Li et al., 2019).

Behavioral experiments to test animal depression include the sucrose preference test and forced swim test. The former reflects the lack of reward stimulation in rats by the quantity of sucrose water that the rats voluntarily consume. The latter involves placing rats in the water and assessing the severity of depression-like behavior according to how long it takes for them to become immobile, in a state of despair, after struggling in the water. In the current study, the sucrose preference time for the exposure group was lower than that for the control group, while the resting time during forced swimming was longer, suggesting that NP caused depression in the rats and led to depression-like behavior.

Monoamine neurotransmitters are excitatory transmitters in the human central nervous system and include DA, NE, and E, which are catecholamines, and the indoleamine 5-HT. DA is a key neurotransmitter in the hypothalamus and pituitary gland, directly affecting human emotions, and causing excitement. The monoamine neurotransmitter hypothesis is the theoretical basis for clinical treatment of patients with depression as well as drug development (Hill, Chopra & Richardi, 2012; Mi & Gao, 2019). It states that the occurrence of depressive symptoms is induced by a decreased NE level in the nervous system. Depletion of NE can induce depression, and an increase in NE can relieve depression.

Our study found that serum catecholamine neurotransmitter levels in the depressed group were significantly lower than those in the healthy group, and the NP exposure group in the animal experiment exhibited higher NP levels than those in the control group, which indicates consistent results between human and animal experiments. The results of this study were also in accordance with the monoamine hypothesis (Li etal., 2017). Spearman correlation analysis indicated that the depression score was negatively correlated with NE and E, and positively correlated with NP level, suggesting that NP may be involved in depression by inhibiting epinephrine, a neurotransmitter.

5-HT, also known as serotonin, is produced by the hydroxylation of tryptophan, and participates in the regulation of mood in the brain together with other central neurotransmitters. Reduced release of 5-HT can induce depression (He et al., 2014). The serum 5-HT levels in the depressed patients in this study were slightly lower than those of the control without significant difference, which was inconsistent with the conclusion we formulated regarding the animal experiment in this study (Jennifer etal., 2013).

BDNF is one of the most important neurotrophic factors, and its level in the brain tissue is positively correlated with the serum level. Decreased BDNF levels in the brain tissues of depressed patients may be associated with the decreased plasticity of neurons in the central nervous system, and decreased serum BDNF will lead to a depression-like state. After antidepressant treatment, the BDNF level in the brain tissues increases, promoting neuronal neogenesis in the hippocampus, where it is closely related to emotional changes (Weihong et al., 2019). HE staining of the hippocampus in the animal experiment showed that the neurons in the hippocampus of the exposure group were arranged in a disorderly fashion, and the nuclei were slightly shrunken, suggesting that NP damaged the hippocampus.

Hayley and Kuhlmann et al. found that the level of BDNF may be related to the severity of depression, i.e., the more severe the depressive symptoms, the lower the level of BDNF in the brain (Sohrab et al., 2018). Our study found that the depression score was negatively correlated with BDNF. Our animal experimental results showed that the levels of BDNF in the NP exposure group were lower than those in the control group. Thus, the results of human and animal experiments were consistent, demonstrating that a reduction in the BDNF level is one possible mechanism for NP causing depression-like behavioral changes.

NP, which is an EED, widely exists in daily life. With the development of industry and urbanization, human exposure to EEDs has increased. EEDs are very soluble in fat. They can enter the blood circulation through food, breathing, and skin absorption, cross the bloodbrain barrier, and subsequently be metabolized and stored by the brain, which is the most fat-rich tissue (60% fat) in the human body. Therefore, the length of time that EEDs remain in the brain tissue is longer as compared to other normal tissues. NP was measured with an average level of 47.8 ng/mL in the serum of all participants in the current study, which is higher when compared to other countries. A previous study showed that the urine levels of NP in adults in the United States, Korea, and Germany were 0.1 ng/mL, 3.7 ng/mL, and 34.4 ng/mL, respectively. According to the results of our NP pharmacokinetics study, only 10% of the dose was excreted through the urine. Because the specimens in this study were serum, the NP levels were higher than other countries (Avinun et al., 2020; Yu et al., 2017). In addition, the results of the previous investigation by our group, where we measured NP in the human body, indicated higher levels. Excluding other factors, age is the main factor, and the current experimental study also confirmed the accumulation of NP in vivo. In the current study, NP accumulation was higher in the depressed group than the healthy group, and there was an association between NP exposure level and SDS score. These findings support the fact that NP has neurotoxic effects, namely, NP plays an important role in the development of depression. Reducing the emission of EEDs in the environment could decrease the risk of depression.

The hospital-based case control survey in this study was carried out with some limitations. First, the sample size was relatively small. Second, the study included the inability of a case control design to determine causation and liability to recall bias.

## Conclusion

In this study, serum NP and monoamine neurotransmitters of depressed and healthy people were measured and analyzed. Combined with animal experiments, it was found that serum NP levels in depressed patients were higher than those in healthy people. The depression scores of both groups were positively correlated with the NP level. From animal experiments, we discovered that chronic exposure to NP at environmental concentrations resulted in the accumulation of NP in the brain and blood, which induced the occurrence of depression in rats, and might be associated with the alterations in the expression levels of BDNF and monoamine neurotransmitters.

Therefore, we concluded that chronic exposure to NP was associated with depression. Our study provides some experimental data regarding the occurrence and development of depression. There should be community-based routine screening for depression among ordinary residents in China. Furthermore, we recommend the creation of support groups for those with psychological disorders as well as counseling facilities within the communities.

##  Supplemental Information

10.7717/peerj.11384/supp-1 Supplemental Information 1 Reagents supplementary material

10.7717/peerj.11384/supp-2 Supplemental Information 2 Vertebrate Animal Study Method

10.7717/peerj.11384/supp-3 Supplemental Information 3 Author Checklist

10.7717/peerj.11384/supp-4 Supplemental Information 4 Human investigation raw data

10.7717/peerj.11384/supp-5 Supplemental Information 5 Water maze animal raw data

## Additional Information and Declarations

### Data Availability

The following information was supplied regarding data availability: Raw data are provided as Supplementary Files.
