# Supplementary material for: Hospital-based case control study and animal study on the relationship between nonylphenol exposure and depression
Source: PeerJ. 2021 May 18;9:e11384. doi: 10.7717/peerj.11384 (PMC8139269; doi:10.7717/peerj.11384)
Supplement: Supplemental Information 1 [file peerj-09-11384-s001.pdf]

Reagents

The nonylphenol (99 % purity) was purchased from the Ehrenstorfer GmbH (Augsburg, Germany). The n-hexane (analytical grade) was purchased from the Jinshan Chemical Reagent Co., Ltd (Chengdu, China). The acetonitrile (chromatographic grade) was purchased from the Tedia Company, (Cincinnati, USA). The Monoamine neurotransmitter kit and 5-HT kit were purchased from the Labor Diagnostika Nord (LDN, Nordhorn, Germany). Hematoxylin and eosin dye was purchased from the Biossci (Hubei) biotechnologies Co., Ltd (Hubei, China). The L500 low-speed electric homogenizer was purchased from Hunan Xiangyi Centrifuge Co. LTD (Hunan, China). The automatic biochemical analyzer Dimension RXL Max was purchased from the Siemens Co., Ltd (Erlangen, Germany). BD53 Fluorescence Microscope was purchased from Olympus (Tokyo, Japan). Microplate reader was purchased Bio-Rad (Richmond, CA). Animal behavior was analyzed using the Noldus EthoVision XT7 software in Morris water maze. All other chemicals were commercially available. All chemical purities were at least 99%.
