# Supplementary material for: Hospital-based case control study and animal study on the relationship between nonylphenol exposure and depression
Source: PeerJ. 2021 May 18;9:e11384. doi: 10.7717/peerj.11384 (PMC8139269; doi:10.7717/peerj.11384)
Supplement: Supplemental Information 2 [file peerj-09-11384-s002.docx]

Four-week-old Sprague-Dawley (SD) male rats were purchased from Changsha Tianqin Biotechnology Co., Ltd. and raised in a clean-grade animal room. Food and water were provided ad libitum at temperature of 24±2°C and humidity of 50% ±10%. After one week of acclimation, 20 rats were randomly divided into the control group and exposure group (n = 10 per group) according to body weight. The exposure group was given an NP dose of 4 mg/kg•bw/day by gavage, and the control group was gavaged with the same volume of corn oil. After 180 days of exposure, the rats were anesthetized with urethane, and the brains were harvested from the abdominal aorta.
